# Furanocoumarin Notopterol: Inhibition of Hepatocellular Carcinogenesis through Suppression of Cancer Stemness Signaling and Induction of Oxidative Stress-Associated Cell Death

**DOI:** 10.3390/nu15112447

**Published:** 2023-05-24

**Authors:** Ting-Yun Huang, Ching-Kuo Yang, Ming-Yao Chen, Vijesh Kumar Yadav, Iat-Hang Fong, Chi-Tai Yeh, Yih-Giun Cherng

**Affiliations:** 1 Department of Emergency Medicine, Shuang-Ho Hospital, Taipei Medical University, New Taipei City 23561, Taiwan; 19123@s.tmu.edu.tw; 2 Graduate Institute of Injury Prevention and Control, College of Public Health, Taipei Medical University, Taipei City 11031, Taiwan; 3 Department of Emergency Medicine, School of Medicine, Taipei Medical University, Taipei City 11031, Taiwan; 4 Division of Colorectal Surgery, Department of Surgery, Mackay Memorial Hospital, Taipei City 10449, Taiwan; yangchingkao@yahoo.com.tw; 5 Division of Gastroenterology and Hepatology, Department of Internal Medicine, School of Medicine, College of Medicine, Taipei Medical University, Taipei City 11031, Taiwan; 08350@s.tmu.edu.tw (M.-Y.C.); vijeshp2@gmail.com (V.K.Y.); 18149@s.tmu.edu.tw (I.-H.F.); 6 Division of Gastroenterology and Hepatology, Department of Internal Medicine, Shuang Ho Hospital, New Taipei City 235041, Taiwan; 7 Continuing Education Program of Food Biotechnology Applications, College of Science and Engineering, National Taitung University, Taitung 95092, Taiwan; 8 Department of Anesthesiology, Shuang Ho Hospital, Taipei Medical University, New Taipei City 23561, Taiwan; 9 Department of Anesthesiology, School of Medicine, College of Medicine, Taipei Medical University, Taipei City 11031, Taiwan

**Keywords:** hepatocellular carcinoma, HCC, notopterol, anticancer treatment, oxidative stress, endoplasmic reticulum stress, cancer stemness

## Abstract

Background: Hepatocellular carcinoma (HCC) remains an aggressive malignancy with a poor prognosis and a leading cause of cancer-related mortality globally. Cumulative evidence suggests critical roles for endoplasmic reticulum (ER) stress and unfolded protein response (UPR) in chronic liver diseases. However, the role of ER stress in HCC pathogenesis, aggressiveness and therapy response remains unclear and understudied. Objectives: Against this background, the present study evaluated the therapeutic efficacy and feasibility of notopterol (NOT), a furanocoumarin and principal component of *Notopterygium incisum*, in the modulation of ER stress and cancer stemness, and the subsequent effect on liver oncogenicity. Methods: An array of biomolecular methods including Western blot, drug cytotoxicity, cell motility, immunofluorescence, colony and tumorsphere formation, flow-cytometric mitochondrial function, GSH/GSSG ratio, and tumor xenograft ex vivo assays were used in the study. Results: Herein, we demonstrated that NOT significantly suppresses the viability, migration, and invasion capacity of the human HCC HepJ5 and Mahlavu cell lines by disrupting ATF4 expression, inhibiting JAK2 activation, and downregulating the GPX1 and SOD1 expression in vitro. NOT also markedly suppressed the expression of vimentin (VIM), snail, b-catenin, and *N*-cadherin in the HCC cells, dose-dependently. Treatment with NOT significantly attenuated cancer stem cells (CSCs)-like phenotypes, namely colony and tumorsphere formation, with the concomitant downregulation of stemness markers OCT4, SOX2, CD133, and upregulated PARP-1 cleavage, dose-dependently. We also demonstrated that NOT anticancer activity was strongly associated with increased cellular reactive oxidative stress (ROS) but, conversely, reduced mitochondrial membrane potential and function in the HepJ5 and Mahlavu cells in vitro. Our tumor xenograft studies showed that compared with sorafenib, NOT elicited greater tumor growth suppression without adverse changes in mice body weights. Compared with the untreated control and sorafenib-treated mice, NOT-treated mice exhibited markedly greater apoptosis ex vivo, and this was associated with the co-suppression of stemness and drug-resistance markers OCT4, SOX2, ALDH1, and the upregulation of endoplasmic reticulum stress and oxidative stress factors PERK and CHOP. Conclusions: In summary, we demonstrated for the first time that NOT exhibits strong anticancer activity via the suppression of cancer stemness, enhanced endoplasmic reticulum stress and increased oxidative stress thus projecting NOT as a potentially effective therapeutic agent against HCC.

## 1. Introduction

Regardless of the remarkable advances made in hepatocellular carcinoma (HCC) diagnostics and treatment strategies, HCC still ranks as the sixth-most-common human malignancy and the third-commonest cause of cancer-related mortality, with over 905,000 new cases and more than 830,000 deaths, respectively, in 2020 alone [1,2]. An increased risk of HCC has been associated with dysregulated signaling pathways, coupled with concomitant oncogene activation/overexpression, and downregulated tumor suppressors in the liver cells; all of these are characteristic of chronic hepatitis B and C (HBV, HCV), addiction to alcohol, dietary toxins, and metabolic liver disease, such as nonalcoholic fatty liver disease (NAFLD) [3,4]. Dysregulated signaling has provided a bedrock for the emergence of targeted therapy as the standard of care for metastatic late-stage HCC in the last decade [5]. Since its approval by the Food and Drug Administration (FDA) over a decade ago, sorafenib has remained the mainstay of treating patients with HCC, until recently with the advent of new generation small molecule inhibitors of tyrosine kinases such as lenvatinib, regorafenib, ramucirumab, and cabozantinib, which have also demonstrated non-inferior therapeutic and/or prognostic effects [5,6]. Nonetheless, HCC remains a fatal malignancy with a high recurrence rate and is often characterized by chemoresistance [3,4,5,6].

The survival benefits of current therapeutic strategies for patients with HCC, including surgical resection and local ablation, are limited by about a 70% 5-year recurrence rate [7]. Unfortunately, resistance to therapy and cancer recurrence after initial treatment remains the greatest causes of HCC morbidity and mortality [7,8], and these cannot be dissociated from the activities of cancer stem cells (CSCs) [8]. CSCs are a small subpopulation of tumor cells with the intrinsic ability to self-renew, modulate cell differentiation, and enhance tumorigenesis [7,8,9]. Accruing evidence continues to highlight the important role of cancer stemness in the development and progression of HCC [8], and targeting these CSCs is increasingly seen as a potentially effective anticancer therapeutic strategy, including for HCC [8,9].

There is increasing evidence that some HCC environmental risk factors, namely HBV, HCV and alcohol addiction, promote liver carcinogenesis by enhancing oxidative stress [10]. Malignant cells predominantly use reactive oxygen species (ROS) and ROS-associated signaling ensuing from nutrient deprivation and hypoxia, which characterize the permissive tumor microenvironment essential for the development and progression of HCC [10]. Conversely, there are also reports of therapy-induced oxidative burst, or so-called ROS burst, resulting in apoptotic or autophagic cell death [10]. ROS is defined as a group of very reactive molecules that are known to regulate important signaling pathways [11]. ROS accumulation plays a critical role in signaling that drives cell cycle progression and cell proliferation. More so, increased ROS production or accumulation irreversibly alters target cellular macromolecules and results in intracellular damage associated with pathological states, including neurodegenerative diseases, cancer, and cell death [10,11,12]. However, as noted by Perillo et al., “ROS are also able to trigger programmed cell death (PCD)” [11]. The present study exploits the probable interplay between ROS production, oncogenic signaling, and cell death induction as a basis for the therapeutic activity and efficacy of notopterol.

Notopterol (NOT) is a furanocoumarin and a principal bioactive component of *Notopterygium incisum*, a traditional Chinese medicinal herb with wide-spectrum pharmacological activity, including anti-inflammatory, pain-modulating, anti-rheumatism, and anti-hypertensive properties [13,14,15]. Intriguingly, a study has reported the anti-proliferative and pro-apoptotic effects of NOT in blood cancer [15], showing its capacity to interfere with the JAK-STAT or STAT3/NF-κB signaling pathway [13,16], which in turn leads to a decrease in the production of inflammatory cytokines and chemokines [13]. In certain HCC cases, the JAK-STAT signaling is abnormally activated, causing the dysregulation of downstream genes that may control processes such as cell survival, angiogenesis, stemness, immune surveillance, evasion, and metastasis [17]. As a result, exploring the potential of NOT as a therapeutic target for HCC presents a valuable research opportunity. However, it remains unclear if and to what extent NOT inhibits the development and/or progression of solid tumors, and more specifically, HCC. In this present study, the therapeutic effect of NOT was investigated, especially in terms of modulating HCC cancer stem cell (CSCs)-like phenotypes associated with aggressive tumor biology, as well as the mechanism underlying the CSCs-inhibitory and oxidative stress-inducing activities of NOT in HCC as a monotherapy and in comparison, with sorafenib, which is FDA-approved for advanced-stage HCC. From our current understanding and review of the existing literature, this study marks the initial examination of NOT’s anticancer properties in hepatocellular carcinoma (HCC), along with its potential to impact cancer stem cells and oxidative stress signaling within HCC cells.

## 2. Materials and Methods

### 2.1. Cells and Cell Culture

Human liver cancer cell lines HepJ5 and Mahlavu generously provided by Dr. Chi-Tai Yeh (Department of Medical Research, Shuang Ho Hospital, New Taipei City, Taiwan) were cultured in Dulbecco’s modified eagle medium (DMEM) supplemented with 10% fetal bovine serum (FBS) and 1% penicillin-streptomycin at 37 °C in a 5% humidified CO_2_ incubator. Cells were sub-cultured at a cell confluence ≥ 95% or *the* medium was changed every 48 h.

### 2.2. Reagents

Notopterol (99.27% purity; Cat.# HY-N0564), was purchased from MedChemExpress (Monmouth Junction, NJ, USA). Stock notopterol solution (10 mM) dissolved in dimethyl sulfoxide (DMSO) and stored at −80 °C in a dark room was further diluted in a sterile culture medium to the final required concentration (DMSO < 0.1%) immediately before use. Standard sorafenib drug was purchased from Santa Cruz Biotechnology (CAS 284461-73-0). A stock solution of 1 mM dissolved in PBS was stored at −20 °C away from light. Antibodies against ATF-4 (Cat.#ab184909), JAK2 (Cat.#3230), phospho-JAK2 (Cat.#3776) were purchased from Cell Signaling Technology (Beverly, MA, USA), SOD1 (ab13498), BCL2 (ab32124), PARP1 (ab191217), cleaved PARP1 (ab32064) from Abcam plc (Cambridge, UK), β-catenin (sc-133240), *N*-cadherin (sc-8424), VIM (sc-373717), Snail (sc-271977), GAPDH (sc-32233), OCT3/4 (sc-5279), SOX2 (sc-365964), and CD133 (sc-365537) from Santa Cruz Biotechnology Inc. (Santa Cruz, CA, USA). Anti-glutathione antibodies (MAB5310, AB5010) were purchased from Sigma-Aldrich (Merck KGaA, Darmstadt, Germany). Alexa Fluor 647 donkey anti-rabbit IgG and Alexa Fluor 488 donkey anti-rabbit IgG were purchased from Invitrogen (Grand Island, NY, USA). Details of all the antibodies along with the dilutions used for this study are described in Supplementary Table S1.

### 2.3. Western Blotting

Following total protein extraction from the untreated control or treated HepJ5 and Mahlavu cells, protein lysate was loaded onto and separated using sodium dodecyl sulfate-polyacrylamide gel electrophoresis (SDS-PAGE), using the Bio-Rad^©^ Mini-Protean III system (Bio-Rad, Taipei City, Taiwan). Thereafter, the protein blots were transferred to polyvinylidene fluoride (PVDF) membranes using the Trans-Blot Turbo Transfer System (Bio-Rad, Taiwan), and the membranes were blocked with 5% skimmed milk or 5% Pierce^TM^ Bovine Serum Albumin (BSA) in Tris-buffered saline-Tween-20 (TBST) for 1 h, and then incubated in monoclonal primary antibody against ATF-4, JAK2, phospho-JAK2, SOD1, BCL2, PARP1, cleaved PARP1, β-catenin, *N*-cadherin, VIM, Snail, OCT3/4, SOX2, CD133, or GAPDH (dilution 1:1000) at 4 °C overnight. After TBST washing, membranes were incubated with horse-radish peroxidase (HRP)-labelled anti-rabbit/mouse IgG secondary antibody (Santa Cruz Biotechnology Inc., Santa Cruz, CA, USA). Protein bands were detected using the Pierce^TM^ ECL Western blotting substrate (Cat.# 32106, ThermoFisher Scientific Inc., Waltham, MA, USA). Protein band images were captured and analyzed using the UVP Biospectrum Imaging System (Vision Works LS 6.8, Level Biotechnology Inc. New Taipei City, Taiwan).

### 2.4. Cell Viability and Drug Combination Assays

HepJ5 and Mahlavu cells were seeded in supplemented complete cell culture media at a density of 4 × 10^3^ cells/well in triplicates in 96-well plates and incubated at 37 °C in 5% humidified CO_2_ for 24 h before exposure to different concentrations of notopterol for up to 72 h. With the untreated wild-type cells serving as control, cell viability and proliferation were assessed by a sulforhodamine B (SRB) assay kit (ab235935; Abcam plc., Cambridge, UK) following the manufacturer’s instructions. Optical density (OD) was measured at 495 nm wavelength in a SpectraMax microplate reader (Molecular Devices, Kim Forest Enterprises Co., Ltd., New Taipei City, Taiwan).

### 2.5. Real-Time Polymerase Chain Reaction (RT-qPCR)

Total RNA was isolated and purified from the HCC cell lines using the TRIzol^TM^ Plus RNA purification kit (Cat.# 12183555, Invitrogen, ThermoFisher Scientific Inc., Waltham, MA, USA) following the manufacturer’s instructions. Briefly, 1 μg of total RNA was reverse-transcribed to cDNA using the QIAGEN One-step RT-PCR Kit (QIAGEN, Taiwan). The qPCR of transcribed cDNA was performed on the iCycler iQ Real-Time detection system (Bio-Rad, Hercules, CA, USA) for the expression of OCT4, SOX2, ALDH1, SOD1, GSH, GSSG, CHOP, and PERK mRNA, with GAPDH serving as the internal control. The specific primer pairs with a sequence are as follows: GAPDH–5′-GTGGTCTCCTCTGACTTCAAC-3′ (sense) and 5′-TCTCTTCCTCTTGTGCTCTTG-3′ (anti-sense), OCT4–5′-CCTGAAGCAGAAGAGGATCACC-3′ (sense) and 5′-AAAGCGGCAGATGGTCGTTTGG-3′ (anti-sense), SOX2–5′-GCTACAGCATGATGCAGGACCA-3′ (sense) and 5′-TCTGCGAGCTGGTCATGGAGTT-3′ (anti-sense), ALDH1–5′-CGGGAAAAGCAATCTGAAGAGGG-3′ (sense) and 5-GATGCGGCTATACAACACTGGC-3′ (anti-sense), SOD1–5′-GGTGAACCAGTTGTGTTGTCAGG-3′ (sense) and 5′-ATGAGGTCCTGCACTGGTACAG-3′ (anti-sense), GSH–5′-GGCATCAGGAAAACGCTA AG-3′ (sense) and 5′-CCTCGCACTTCTCAAAAAGC-3′ (anti-sense), CHOP–5′-GGAGGCAGAGGTTGCAGTGA-3′ (sense) and 5′-GCTCTGTTGCCAGGCTGAAG-3′ (anti-sense), and PERK–5′-GTCCCAAGGCTTTGGAATCTGTC-3′ (sense) and 5′-CCTACCAAGACAGGAGTTCTGG-3′ (anti-sense). SYBR^TM^ Green I nucleic acid gel stain (Cat.# S7563, Invitrogen, ThermoFisher Scientific Inc., Waltham, MA, USA) was used as a fluorescent dye for PCR product detection according to the manufacturer’s instruction. Cycling conditions were 95 °C for 3 min, followed by 40 cycles at 94 °C for 30 s, 62 °C for 30 s and 72 °C for 1 min. mRNA expression normalization was achieved using GAPDH. Details of all the primers along with the sequence used for this study are described in Supplementary Table S2.

### 2.6. Tumorsphere Formation Assay

Moreover, 1 × 10^3^ untreated control or treated HepJ5 and Mahlavu cells were seeded per well containing 1 mL serum-free DMEM medium supplemented with 20 ng/mL B27 (Invitrogen, ThermoFisher Scientific Inc., Waltham, MA, USA), and 10 ng/mL epidermal growth factor (BD Biosciences, Palo Alto, CA, USA) in 12-well ultra-low attachment plates incubated at 37 °C in 5% humidified CO_2_ incubator. The generated non-adherent spheroids (>90 μm in diameter) were counted, and then the spheroids were measured using an ocular micrometer.

### 2.7. Transwell Matrigel Invasion Assay

Using a 24-well plate transwell system, we evaluated the invasion ability of the cells. The upper chambers of the transwell system were pre-coated with solubilized matrigel (BD Bioscience). After matrigel polymerization, 2 × 10^4^ HepJ5 and Mahlavu cell lines were seeded into each upper chamber insert containing 200 μL FBS-free DMEM with different concentrations of notopterol, while the lower chamber contained 500 μL DMEM with 10% FBS serving as a chemoattractant. The medium was discarded after cell incubation for 24 h. The invaded cells on the lower side of the membrane were fixed with 10% formaldehyde for 20 min at room temperature and then stained with 0.2% (*w*/*v*) crystal violet solution for 20 min. The cells remaining on the upper side of the membrane were carefully removed with a cotton bud. The invaded cells were observed under a microscope and the total number of cells on the lower surface was counted.

### 2.8. Scratch Wound Migration Assay

Untreated control or treated Mahlavu and HepJ5 cells were seeded onto 6-well plates with complete growth media containing 10% FBS, and cultured to 100% confluence. The cell monolayers were scratched with a sterile yellow pipette tip to scratch the culture wells along the median axes, and fresh culture medium with or without notopterol was added. The wound closure migration images were captured at 0 and 48 h after the wound scratch, under a microscope with a 10× objective lens, and analyzed with the NIH ImageJ 149 software were downloaded from https://imagej.nih.gov/ij/download.html (accessed on 12 April 2023) and installed on a local computer.

### 2.9. Colony Formation Assay

Furthermore, 2 × 10^4^ HepJ5 or Mahlavu cells were plated into 6-well cell culture plates and incubated at 37 °C for 2 weeks after treatment with or without notopterol. The HCC cells were then washed 3 times with cold 1× PBS, fixed with ice-cold methanol, stained with crystal violet dye, washed 3 times again with PBS, and dried at room temperature. The colonies formed were then evaluated and counted under a microscope. In each well, the total number of colonies (diameter ≥ 100 μm) was counted over 5 randomly selected fields in triplicate assays.

### 2.10. Hoechst33342 Side-Population (SP) Staining Flow Cytometry Assay

Hoechst33342 staining was used to evaluate the effect of notopterol on HCC side population cells. Cells were seeded at a concentration of 5 × 10^5^ cells/mL and incubated for 48 hrs with the vehicle, notopterol, or sorafenib at indicated concentrations. Cells were harvested and washed thrice with ice-cold 1× PBS. Cells were then re-suspended in DMEM containing 2% FBS at 1 × 10^6^ cells/mL containing Hoechst33342 (Cat.#B2261, Sigma-Aldrich, Merck KGaA, Darmstadt, Germany) at 5 µg/mL alone or combined with 50 µM inhibitor of ABC transporter, verapamil. After 90 min incubation at 37 °C, a BD LSRFortessa Cell Analyzer (BD Biosciences, San Jose, CA, USA) was used to perform flow cytometry analyses, using 488 nm excitation and fluorescence detection in log mode at the nominally far-red wavelengths > 695 nm. Hoechst33342 emission was initially split using a 610 nm dichroic short-pass filter, with red and blue emissions collected through 670/30 nm and 450/65 nm wavelength bandpass filters, respectively. Vehicle-treated and verapamil-treated cells served as the negative and positive control, respectively.

### 2.11. Detection of 2,7-Dichlorodihydrofluoroscin Diacetate (DCFDA) Intracellular Reactive Oxygen Species (ROS) Assay

Intracellular ROS level was measured using the ROS detection cell-based assay kit (DCFH-DA) (Cat.#601520, Cayman Chemical). The quantified principal component was cell-permeable DCFH-DA, which is easily oxidized to fluorescent dichlorofluorescein (DCF) by intracellular ROS. Briefly, HepJ5 or Mahlavu cells, seeded in 96-well plates and treated with indicated concentrations of notopterol for 24 h, were incubated with DCFH-DA at 37 °C for 20 min. The cells were then observed under a fluorescence microscope and measured at 488 nm and 525 nm excitation and emission wavelength, respectively, in a fluorescence microplate spectrophotometer (BioTek, SunPro International Inc., Taipei City, Taiwan).

### 2.12. Detection of Oxidative Stress Using MitoTracker^®^ Green Assay

Oxidative stress was detected using the MitoTracker^®^ Green FM (Cat.# M7514, ThermoFisher Scientific Inc., Waltham, MA, USA) following the manufacturer’s instructions. Briefly, HepJ5 and Mahlavu cells seeded and cultured in 96-well plates were treated with or without indicated concentrations of notopterol for 1 h and then stained with 20 nM MitoTracker^®^ Green for 30 min in a complete cell growth medium. Cells were then washed thrice with cold 1× PBS and imaged under the fluorescence microscope.

### 2.13. Detection of Intracellular Reduced Glutathione (GSH) Level

To measure the intracellular GSH level, the reduced glutathione (GSH) assay kit (colourimetric) (Cat.# MAK364, Sigma-Aldrich, Merck KGaA, Darmstadt, Germany) was utilized. The modified Tietze method, as described previously [18], was employed to determine the GSH levels in cells treated with or without the indicated treatment.

### 2.14. Tumor Xenograft Studies

Fifteen female BALB/c nude mice (6 weeks old, weighing 18–20 g) were purchased from BioLASCO (BioLASCO Taiwan Co., Ltd., Taipei, Taiwan). Animals were used according to protocols approved by the Laboratory Animal Committee of the Taipei Medical University (Protocol LAC-2021-0670). All mice were housed per experimental group in large cages (5 per cage) under specific pathogen-free (SPF) conditions: a temperature of 21 ± 7 °C, a humidity of 55 ± 5%, and a 12 h light/dark cycle with the lights coming on at 7.00 A.M.). The mice were fed a standard meat-free rat and mouse diet (SF00-100, Specialty Feeds, Western Australia, Australia), and freely accessed clean drinking water. The mice were randomly allocated into one of 3 experimental groups, namely control (*n* = 5), 30 μM Sora (*n* = 5), or 30 μM NOT (*n* = 5) for mice inoculated with cells treated with vehicle, 30 μM sorafenib or 30 μM notopterol, respectively, for 24 h before tumor inoculation. Tumor growth was monitored daily, and tumor size was measured every 72 h using callipers and the formula: (x × y^2^)/2, where x = longest diameter, and y = diameter perpendicular to x. Following 4 weeks, the mice were humanely euthanized, and their tumors were collected for analysis according to the standard protocol at the Laboratory Animal Center (LAC) of Taipei Medical University. A vaporizer was used to administer 5% isoflurane as the anaesthetic agent for euthanasia in small animals such as mice.

### 2.15. Data Analysis

Data are presented as means ± SEM (standard error of the mean) of experiments performed at least 3 times in triplicate. Statistical analyses were performed using GraphPad Prism ver. 7.0 for Windows (GraphPad Software, San Diego, CA, USA). Comparison between two groups was performed using a 2-sided Student’s *t*-test, while the comparison of ≥3 groups was performed with the one-way analysis of variance (ANOVA). *p*-value < 0.05 was considered to be statistically significant.

## 3. Results

### 3.1. Notopterol Suppresses Hepatocellular Carcinoma Cell Viability by Inhibiting JAK2 Activation and Enhancing Oxidative Stress

**Figure 1:**
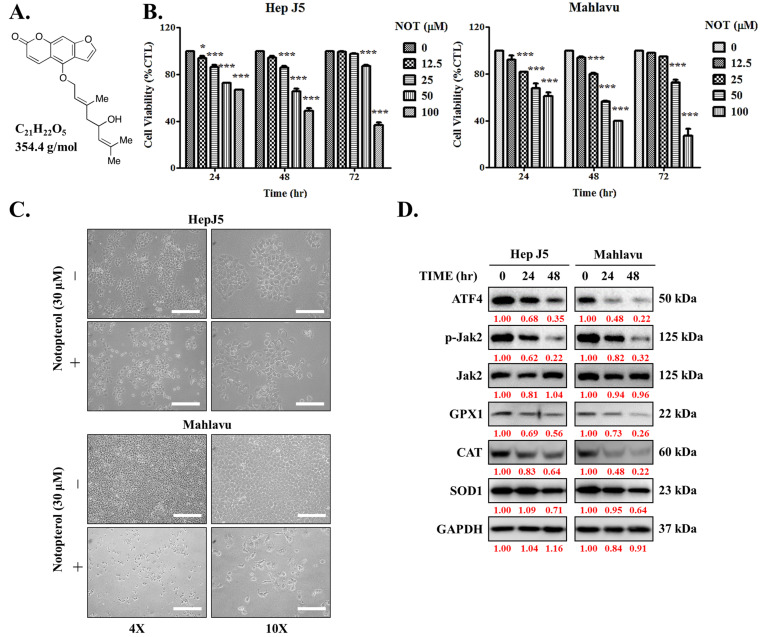
Notopterol suppresses hepatocellular carcinoma cell viability by inhibiting JAK2 activation and enhancing oxidative stress. (**A**) Chemical structure of notopterol with a molecular weight of 354.4 g/mol. (**B**) Line graphs showing the effect of 0–100 μM notopterol on the viability of HepJ5 and Mahlavu cell lines at 24, 48, and 72 h. (**C**) Photo images showing the effect of 48 h treatment with 30 μM notopterol on the viability and morphology of HepJ5 and Mahlavu cell lines at 4× and 10× magnification. Scale bar = 50 μm. (**D**) Representative Western blot images showing the effect of 30 μM notopterol on the expression levels of ATF4, p-JAK2, JAK2, GPX1, CAT and SOD1 in HepJ5 or Mahlavu cell lines at 0, 24, and 48 h. GAPDH was loading control. * *p* < 0.05, *** *p* < 0.001.

First, the impact of notopterol (NOT), which has a depicted chemical structure, molecular formula of C21H22O5, and molecular weight of 354.4 g/mol (Figure 1A), was evaluated on HCC using the HepJ5 and Mahlavu cell lines. The cell cytotoxicity assays of NOT-treated cells compared with untreated control cells revealed that HepJ5 cells exhibited significantly reduced cell viability in a dose- and time-dependent manner. Specifically, exposure to 100 μM NOT for 24 h, 48 h, and 72 h resulted in a 33% (*p* < 0.001), 49% (*p* < 0.001), and 64% (*p* < 0.001) reduction in viability, respectively (Figure 1B, left). Similarly, a 39% (*p* < 0.001), 60% (*p* < 0.001), and 79% (*p* < 0.001) reduction in Mahlavu cell viability was observed upon treatment with 100 μM NOT for 24 h, 48 h, and 72 h, respectively (Figure 1B, right). Upon 48 h treatment with 30 μM NOT, in addition to the significant reduction in cell numbers, a transformation of the HCC cells from elongated spindle-like to cuboid morphology was observed (Figure 1C). In parallel assays, treatment with 30 μM NOT was shown to markedly downregulate the expression levels of ATF4, p-JAK2, GPX1, CAT, and SOD1 proteins in the HepJ5 and Mahlavu cell lines in a time-dependent manner (Figure 1D). These results suggest that notopterol suppresses HCC cell viability by inhibiting JAK2 activation and enhancing oxidative stress through the modulation of the aforementioned markers.

### 3.2. Notopterol Significantly Attenuates HCC Cell Migration and Invasive Capacity

**Figure 2:**
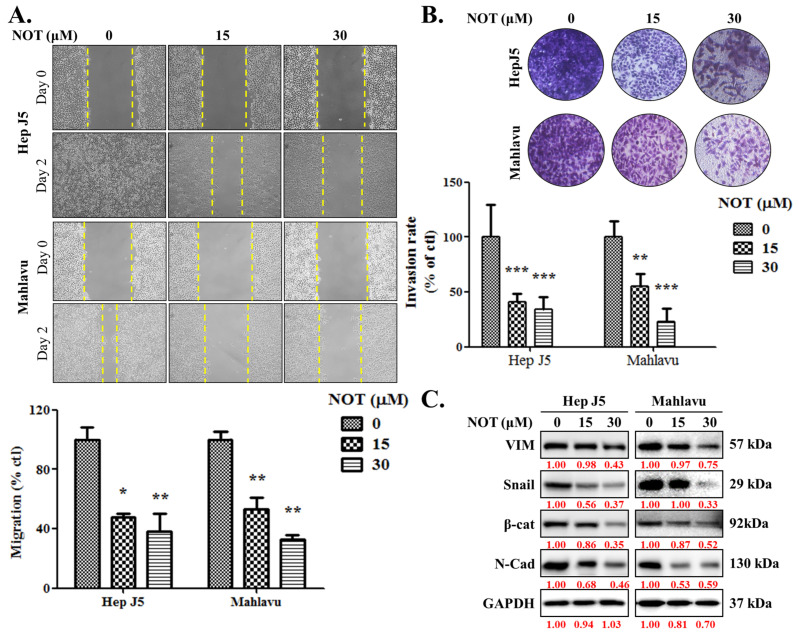
Notopterol significantly attenuates HCC cell migration and invasive capacity. Photo-images and histograms of the effect of 0–30 μM notopterol on the (**A**) migration and (**B**) invasion capabilities of HepJ5 and Mahlavu cell lines at Day 0 and Day 2 after wound-scratch. (**C**) Representative Western blot images showing the effect of 0–30 μM notopterol on the expression levels of VIM, Snail, β-catenin, and *N*-cadherin in HepJ5 or Mahlavu cell lines at 0, 24, and 48 h. GAPDH was loading control. VIM, vimentin; β-cat, β-catenin; *N*-cad; *N*-cadherin. * *p* < 0.05, ** *p* < 0.01, *** *p* < 0.001.

As enhanced cell migration and invasion are key components of cancer metastasis and progression [19], the effect of NOT on these aspects was investigated. When compared with the untreated control, HepJ5 cells treated with 15 and 30 μM NOT exhibited a 53% (*p* < 0.05) and 62% (*p* < 0.01) decrease in migration, respectively (Figure 2A). A 47% (*p* < 0.01) and 68% (*p* < 0.01) reduction in migration was observed in Mahlavu cells treated with 15 and 30 μM NOT, respectively (Figure 2A). Similarly, treatment with 15–30 μM NOT significantly inhibited the invasion of HepJ5 (56–79%, *p* < 0.001) and Mahlavu (52–81%, *p* < 0.001) cells compared to the untreated control cells (Figure 2B). Additionally, it was found that HepJ5 and Mahlavu cells treated with 15–30 μM NOT displayed a significant dose-dependent downregulation of VIM, Snail, β-catenin, and *N*-cadherin protein expression levels (Figure 2C).

### 3.3. Notopterol Effectively Inhibits the Cancer Stem Cells (CSCs)-Like Phenotypes of HCC Cells

**Figure 3:**
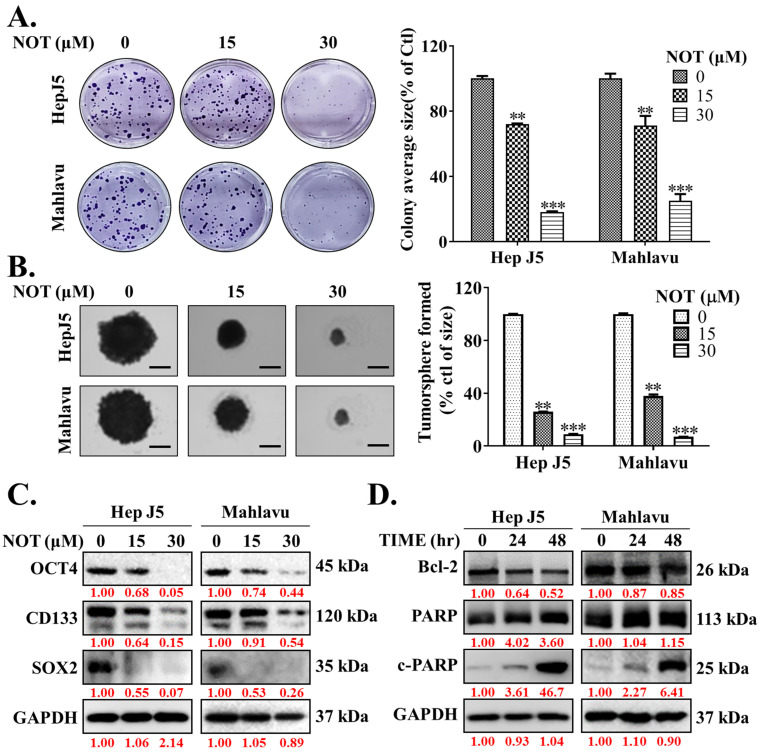
Notopterol effectively inhibits the cancer stem cells (CSCs)-like phenotypes of HCC cells. Photo-images and histograms of the effect of 0–30 μM notopterol on the (**A**) colony formation and (**B**) tumorsphere formation capabilities of HepJ5 and Mahlavu cell lines. (**C**) Representative Western blot images showing the effect of 0–30 μM notopterol on the expression levels of OCT4, CD133, and SOX2 in HepJ5 or Mahlavu cell lines. (**D**) Representative Western blot images showing the effect of 30 μM notopterol on the expression levels of BCL-2, PARP, and c-PARP in HepJ5 or Mahlavu cell lines after 24 or 48 h treatment, compared with untreated control. GAPDH was loading control. c-PARP, cleaved PARP. ** *p* < 0.01, *** *p* < 0.001.

Ample evidence suggests that cancer stem cells (CSCs) drive the development and growth of cancers and are associated with increased tumor aggressiveness, therapy resistance, secondary site colonization, and cancer recurrence [20]. Consequently, the potential effect of NOT on the CSCs-like phenotypes of HCC cells was evaluated. The results demonstrated that NOT significantly reduced the ability of HepJ5 (15 μM: 28%, *p* < 0.01; 30 μM: 82%, *p* < 0.001) and Mahlavu (15 μM: 29%, *p* < 0.01; 30 μM: 75%, *p* < 0.001) cells to form colonies (Figure 3A). Furthermore, treatment with 15-30 μM NOT substantially decreased the number and sizes of tumorspheres formed by HepJ5 (68–84%, *p* < 0.001) and Mahlavu (61–87%, *p* < 0.001) cells (Figure 3B). Interestingly, 15–30 μM NOT was also found to markedly downregulate the expression levels of OCT4, CD133, and SOX2 proteins in HepJ5 and Mahlavu cells (Figure 3C). Moreover, treatment with 30 μM NOT for 48 h suppressed the expression of BCL-2 protein, while simultaneously upregulating cleaved PARP-1 (c-PARP-1) protein expression level in a time-dependent manner (Figure 3D).

### 3.4. Notopterol Anticancer Activity Is Mediated by Increased Intracellular Reactive Oxygen Species (ROS) Activity, Mitochondrial Dysfunction and Oxidative Stress Induction in HCC Cells

**Figure 4:**
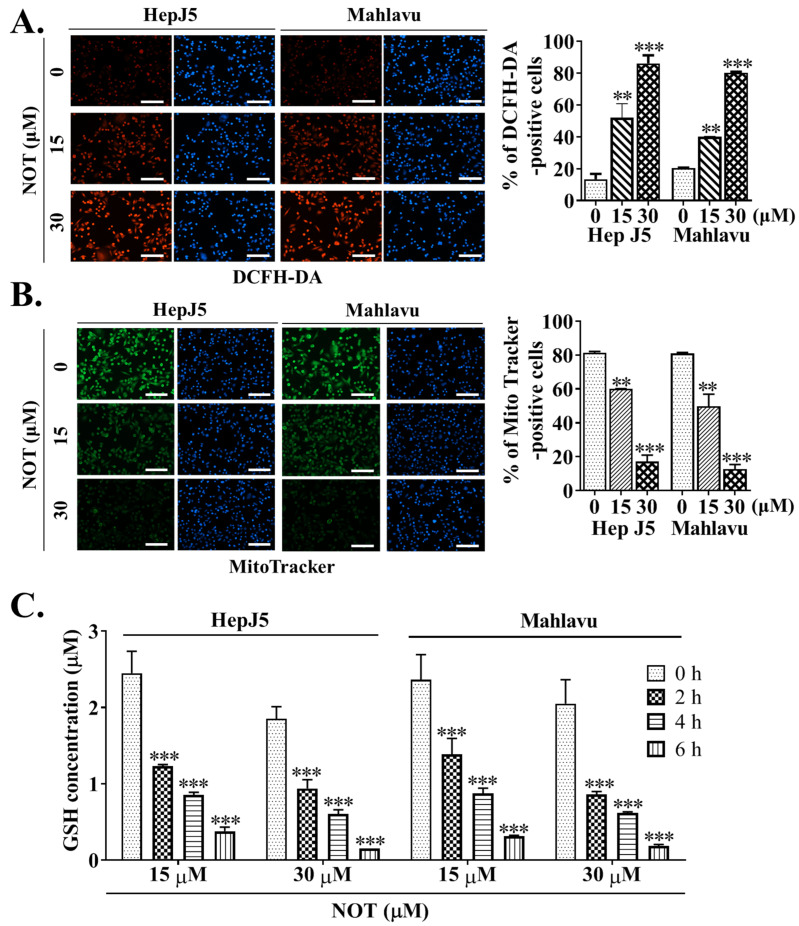
Notopterol anticancer activity is mediated by increased intracellular reactive oxygen species (ROS) activity, mitochondrial dysfunction and oxidative stress induction in HCC cells. Photo-images and histograms showing the (**A**) fluorescence signal of DCFH-DA (red) and (**B**) MitoTracker staining (green) in HepJ5 or Mahlavu cell lines treated with 0–30 μM NOT. DAPI (blue) served as a nuclear marker. (**C**) Histograms showing the effect of 0–6 h treatment with 15 or 30 μM NOT on GSH concentration in HepJ5 and Mahlavu cell lines. NOT, notopterol; GSH, reduced glutathione; ** *p* < 0.01, *** *p* < 0.001.

Dichlorodihydrofluorescein diacetate (DCFH-DA) is widely used in detecting reactive oxygen species (ROS) activity. In comparison to untreated HCC cells, treatment with 15 and 30 μM NOT resulted in enhanced ROS activity, as demonstrated by a 32% (*p* < 0.01) and 44% (*p* < 0.001) increase in DCFH-DA positive HepJ5 cells, or a 29% (*p* < 0.01) and 41% (*p* < 0.001) increase in Mahlavu cells, respectively (Figure 4A). Considering that the mitochondrial mass stain, MitoTracker^®^ green (MTG), is positively correlated with ROS and reactive nitrogen species (RNS) markers [21], it was further shown that, compared to the brighter fluorescence of active mitochondria in untreated control HCC cells, NOT treatment reduced the mitochondrial fluorescence intensity and the number of MitoTracker^®^-positive HepJ5 (15 μM: 37%, *p* < 0.01; 30 μM: 76%, *p* < 0.001) or Mahlavu (15 μM: 33%, *p* < 0.01; 30 μM: 64%, *p* < 0.001) cells. This suggests enhanced ROS production, increased mitochondrial degeneration, and impaired biogenesis (Figure 4B). Additionally, it was demonstrated that treating HepJ5 and Mahlavu cells with 15 and 30 μM NOT for 6 h significantly reduced the antioxidant glutathione (GSH) level compared to untreated control counterparts, in a dose- and time-dependent manner (*p* < 0.001) (Figure 4C).

### 3.5. Notopterol Inhibit HCC Tumor Development and Growth by Disrupting Stemness Signals with Endoplasmic Reticulum and Oxidative Stress Induction, Ex Vivo

**Figure 5:**
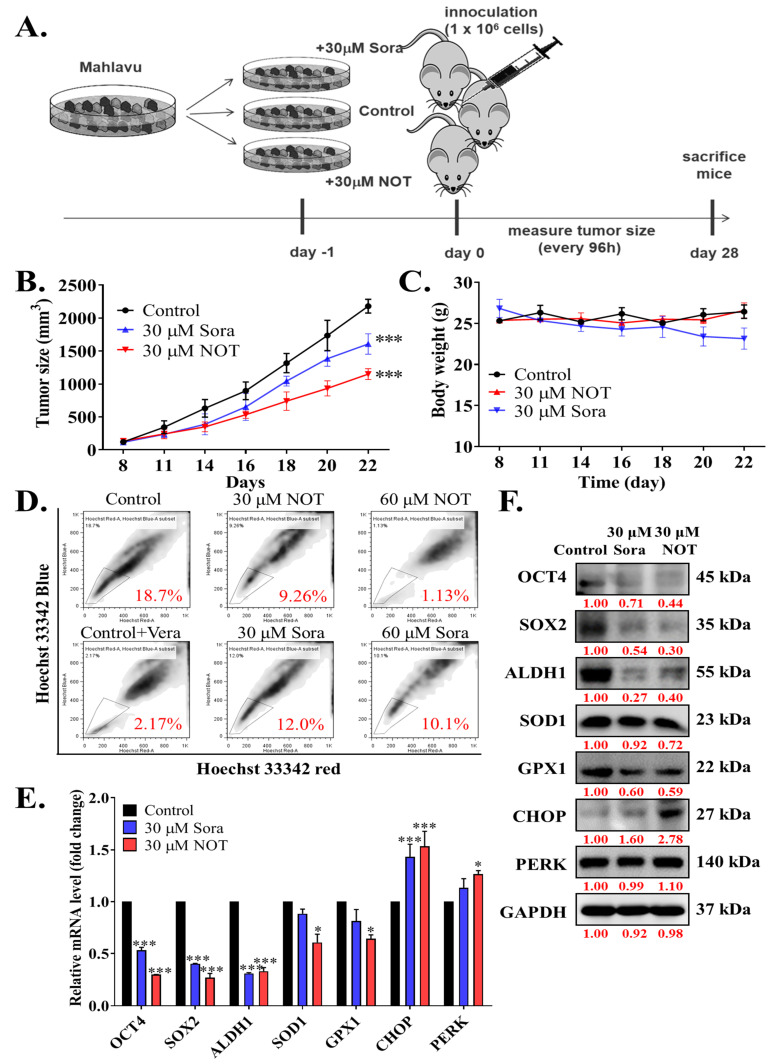
Notopterol inhibits HCC tumor development and growth by disrupting stemness signals with endoplasmic reticulum and oxidative stress induction, ex vivo. (**A**) Schema of the mice HCC tumor xenograft experimental design using BALB/c nude mice inoculated with Mahlavu cells pre-treated with 30 μM NOT or 30 μM Sora. Line graphs comparing the effects of 30 μM NOT and 30 μM Sora on the (**B**) tumor sizes, and (**C**) changes in body weights of xenografted mice over 22 days. (**D**) Flow cytometry analysis showing the effect of pre-treatment with 30 μM NOT or 30 mM Sora on Hoechst 33342 staining in cells dissociated from tumors harvested from the 30 μM NOT or 30 μM Sora xenografted mice group, in the absence or presence of Vera. The SP cells were gated and shown as a percentage as indicated. (**E**) Histograms showing the effect of pre-treatment with 30 μM NOT or 30 μM Sora on OCT4, SOX2, ALDH1, SOD1, GPX1, CHOP, or PERK mRNA expression levels. (**F**) Representative Western blot images showing the effect of 30 μM NOT and 30 μM Sora on the expression levels of OCT4, SOX2, ALDH1, SOD1, GPX1, CHOP and PERK in an ex vivo mice model. GAPDH was loading control. Sora, sorafenib; NOT, notopterol; Vera, verapamil; SP, side-population; * *p* < 0.05, *** *p* < 0.001.

After demonstrating the anticancer effect of NOT in vitro, these findings were further validated by monitoring xenografted tumor formation in female BALB/c nude mice subcutaneously inoculated with or without treated 1 × 10^6^ Mahlavu cells. Mice were randomly divided into three experimental groups, namely control (*n* = 5), 30 μM Sora (*n* = 5), or 30 μM NOT (*n* = 5) for cells treated with vehicle, 30 μM sorafenib or 30 μM notopterol, respectively, for 24 h (Figure 5A). Sorafenib, a first-line therapeutic drug currently approved for use, served as the positive control in this experiment. Our results showed that compared to the control mice, tumors were smaller and the tumor growth rate was lower in 30 μM Sora and 30 μM NOT groups with 1.47-fold (*p* < 0.001) and 2.16-fold (*p* < 0.001) smaller tumors, respectively, by day 22 post-inoculation (Figure 5B). Unlike the 30 μM Sora mice, mice in the 30 μM NOT group exhibited no adverse loss or increase in body weight (Figure 5C). Side population (SP) cells, enriched with CSCs, are known to exhibit a distinct ability to actively efflux Hoechst33342 dye from the cytoplasm through verapamil-sensitive ATP-binding cassette (ABC) transporters [22]. Interestingly, an ex vivo Hoechst 33342 SP assay using cells dissociated from harvested tumors from the three mice groups showed that compared with the negative control (18.7% SP cells), 30 μM Sora mice exhibited a 1.56-fold reduction in the number of SP cells, while the 30 μM NOT mice exhibited the least amount of SP cells (2.02-fold reduction) (Figure 5D). In parallel assays using mice inoculated with Mahlavu cells pre-treated with 60 μM Sora or 60 μM NOT, a substantially diminished level of SP cell population was observed in the presence of verapamil, an inhibitor of ABC transporter (8.62-fold reduction). However, the 60 mM NOT mice exhibited even more significant inhibition of SP cells (16.5-fold reduction), while a minimal 1.85-fold reduction was observed in the 60 mM Sora mice (Figure 5D). Notably, it was demonstrated that, compared with the control, in tumors from the 30 mM Sora or 30 mM NOT mice, the expression levels of OCT4 (Sora: 0.46-fold, *p* < 0.001; NOT: 0.65-fold, *p* < 0.001), SOX2 (Sora: 0.53-fold, *p* < 0.001; NOT: 0.79-fold, *p* < 0.001), ALDH1 (Sora: 0.63-fold, *p* < 0.001; NOT: 0.65-fold, *p* < 0.001), SOD1 (Sora: 0.07-fold, *p* < 0.05; NOT: 0.42-fold, *p* < 0.05), GPX1 (Sora: 0.46-fold, *p* < 0.05; NOT: 0.65-fold, *p* < 0.05) at mRNA level were markedly downregulated. Conversely, CHOP (Sora: 1.33-fold, *p* < 0.001; NOT: 1.53-fold, *p* < 0.001) and PERK (Sora: 1.21-fold, *p* < 0.05; NOT: 1.45-fold, *p* < 0.05) mRNA levels were significantly upregulated (Figure 5E). Additionally, the protein expression levels of OCT4 (Sora: 0.71-fold, *p* < 0.001; NOT: 0.44-fold, *p* < 0.001), SOX2 (Sora: 0.54-fold, *p* < 0.001; NOT: 0.30-fold, *p* < 0.001), ALDH1 (Sora: 0.27-fold, *p* < 0.001; NOT: 0.40-fold, *p* < 0.001), SOD1 (Sora: 0.92-fold, *p* < 0.05; NOT: 0.72-fold, *p* < 0.05), and GPX1 (Sora: 0.60-fold, *p* < 0.05; NOT: 0.59-fold, *p* < 0.05) were notably downregulated. In contrast, CHOP (Sora: 1.60-fold, *p* < 0.05; NOT: 2.78-fold, *p* < 0.001) and PERK (Sora: 0.99-fold, *p* < 0.01; NOT: 1.1-fold, *p* < 0.001) at protein levels were significantly upregulated (Figure 5F).

## 4. Discussion

HCC remains a fatal malignancy with a high recurrence rate and is often characterized by chemoresistance [3,6,22]. The characteristic HCC aggressive phenotypes, including chemoresistance, recurrence, and metastasis have been attributed to the presence of hepatocellular CSCs [7,8,9,22]. Hepatocellular CSCs essentially exhibit the capability to de-differentiate and self-renew and are implicated in the development and progression of HCC based on expressed specific cell surface markers, pluripotency biomarkers, and constitutively upregulated aldehyde dehydrogenase (ALDH) activity in HCC [23,24,25]. These hepatocellular CSCs often exhibit a quiescent phenotype with an intrinsic propensity for enhanced cell viability, self-renewal and cellular longevity by modulating oxidative stress markers, cancer stemness factors, and related biomarkers of cancer aggression, and thus the heightened interest in oxidative stress and CSCs as therapeutic targets in patients with metastatic HCC.

In the present study, it was demonstrated that notopterol suppresses hepatocellular carcinoma cell viability by inhibiting JAK2 activation and enhancing oxidative stress (Figure 1). This is consistent with recent work by Wang Q et al. demonstrating that NOT ameliorates inflammation and rheumatoid arthritis by directly binding to the kinase domain of JAK2 and thereby inhibiting the Janus kinase 2/signal transducer and activator of transcription 3 (JAK2/STAT3) signaling pathway [13]. Contextually, the aberrant activation of JAK2/STAT3 signaling characterizes various malignancies and is implicated in the tumorigenesis, angiogenesis, metastasis, and recurrence of many cancers, including HCC, and particularly those metastatic and/or recurrent cancers that are refractory to the standard chemotherapy [26,27]. It is clinically interesting that NOT not only inhibits JAK2 activation but that it enhances intracellular ROS-related oxidative stress. It is not impossible that by facilitating increased ROS flux, NOT elicits irreversible alteration of target macromolecules in the liver cancer cells, thereby inducing bio-cellular damage within the malignant hepatic cells, which consequently leads to a persistent redox homeostasis disequilibrium that culminates in HCC cell death. The glutathione redox cycle is a principal intracellular defence system consisting of GSH, which acts as a ROS scavenger and regulates the intracellular redox state, alongside glutathione peroxidase (GPx) and glutathione reductase (GR). The ability of any cell, and in this case, liver cancer cells, to regenerate GSH from its oxidized form GSSG is fundamental in buffering oxidative stress [10,11]; however, to the best of our current knowledge, this study demonstrated for the first time that NOT upregulated GSSG at the expense of GSH, thereby eliciting enhanced oxidative stress.

This study also showed that notopterol significantly attenuates HCC cell migration and invasive capacity, as well as effectively inhibits the cancer stem cells (CSCs)-like phenotypes of HCC cells (Figure 2 and Figure 3). This is of clinical relevance, especially as the contemporary literature reports the strong association between hepatocellular CSCs, the development of HCC, and increased malignant traits of HCC such as enhanced invasiveness, resistance to treatment, early recurrence, easy metastasis, and poor prognosis [9,27,28]. The findings are consistent with the current knowledge that the plasticity of CSCs and the promotion of oncogenic activities such as migration and invasion, which are essential for HCC metastasis, more so as CSCs markers and the epithelial-to-mesenchymal transition (EMT), are closely associated with HCC metastasis [9,27,28]. It is postulated that by suppressing JAK2 phosphorylation, NOT inhibits JAK2/STAT3 signaling, enhances intracellular ROS burst, and destabilizes VIM, Snail, β-catenin, or *N*-cadherin oncogene interaction with the stemness marker SOX2 or OCT4, thus suppressing tumorsphere formation and oncogenicity and consequently leading to HCC cell death as expressed by upregulated cleaved PARP levels. This would be consistent with the demonstrated NOT-elicited inhibition of JAK2 activation, and the documented roles of JAK2/STAT3 signaling in oncogenicity, maintenance of cancer stemness, cellular survival under stress, and resistance to multiple anticancer therapies [8,9,10,11,24,25,26,27,28,29].

Furthermore, to the best of our knowledge, our study demonstrates for the first time that notopterol anticancer activity is mediated by disrupted stemness signals, increased intracellular ROS activity, and oxidative stress induction in HCC cells, in vitro and ex vivo (Figure 4 and Figure 5). ROS acts as an important signaling molecule that tightly regulates CSCs plasticity and fate through the modulation of several intersecting intracellular signaling pathways [30]. Our findings corroborate the contemporary understanding that compared to normal cells, malignant cells may be more sensitive to ROS accumulation. Though rapid increases in intracellular ROS have been suggested to cause cellular transformation and tumorigenesis [29,30,31], in this study, it was demonstrated that NOT #stantially enhanced the ROS flux in HCC cells and that this substantial intracellular accumulation of ROS elicits irreversible cellular damage and consequent death. Interestingly, it was shown that the therapeutic potential of NOT was superior to that of sorafenib in terms of inhibiting side population or CSCs activity, inducing oxidative stress within malignant liver cells, and preventing tumor growth ex vivo (Figure 5). Sorafenib, which is currently approved as a first-line treatment drug, was used as the positive control in this study. It functions by inhibiting the growth of tumor cells and the formation of blood vessels through targeting various serine/threonine and tyrosine kinases such as RAF1, BRAF, VEGFR 1, 2, 3, PDGFR, KIT, FLT3, FGFR1, and RET [32]. These are key players in several oncogenic signaling pathways. However, most patients eventually develop resistance to it. The ability of NOT to harness these biological features of ROS suggests the potential use of NOT as an effective ROS-mediated, CSCs-targeting, anticancer therapeutic agent for the treatment of patients with HCC, and that NOT can overcome therapy resistance.

**Figure 6:**
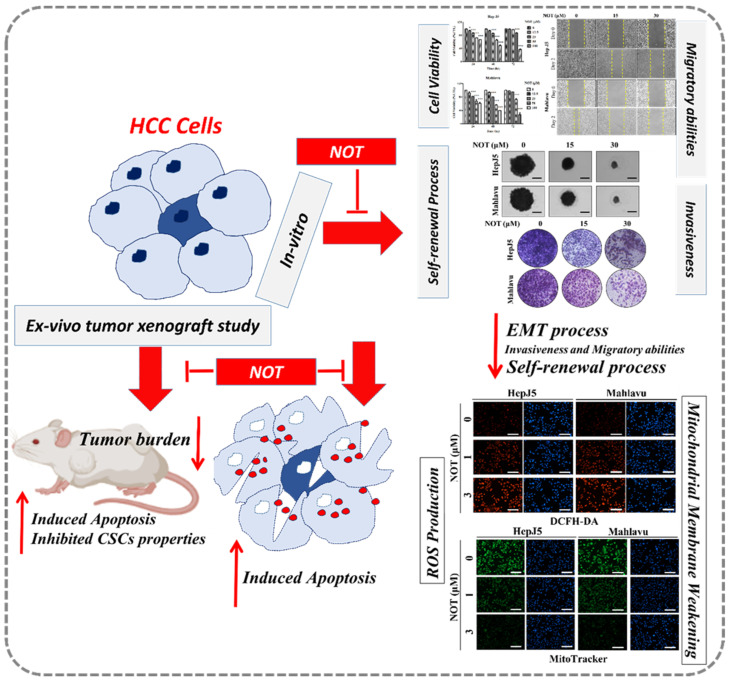
In the schematic figure, we demonstrated for the first time that NOT exhibits strong anticancer activity in vitro and ex vivo via suppression of cancer stemness, enhanced endoplasmic reticulum stress and increased oxidative stress.

In conclusion, as shown in the schematic of Figure 6, the findings presented herein are clinically relevant for HCC management because of the prevalent tendency of HCC cells to resist contemporary chemotherapeutic agents, as exemplified by a 0.25 response rate, no significant increase in patient overall survival [33], which is invariably associated with permissive TME, and the presence of hepatocellular CSCs, as demonstrated in our study. The present study, therefore, reports an effective anti-HCC therapeutic strategy that selectively kills cancerous liver cells, based on CSCs targeting and increased oxidative stress by exogenous ROS generation therapy. In this study, it was demonstrated that NOT effectively moderates the ROS environment in HCC cells both in vitro and ex vivo, and targets key CSCs markers via the modulation of oxidative stress. However, many key factors, such as the role of miRNAs in epigenetically controlling CSCs and ROS, as well as NOT’s role in this process, have not been studied to uncover the molecular mechanism of NOT in overcoming the therapeutic challenge in HCC. Therefore, in future research, the aim will be to uncover the epigenetic role of key molecules in HCC, which may be modulated by the introduction of NOT, and to reveal the key molecular mechanisms of NOT in overcoming the therapeutic challenge.

## Data Availability

The datasets used and analyzed in the current study are publicly accessible as indicated in the manuscript.

## Supplementary Materials

The following are available online at https://www.mdpi.com/article/10.3390/nu15112447/s1, Supplementary Table S1. The membranes were incubated in primary antibodies. Supplementary Table S2. List of gene-specific primers with detailed sequence used in this study. Supplementary Figure S1. Full Western-blotting result of the representative image in Figure 1D. Supplementary Figure S2. Full Western-blotting result of the representative image in Figure 2C. Supplementary Figure S3. Full Western-blotting result of the representative image in Figure 3C. Supplementary Figure S4. Full Western-blotting result of the representative image in Figure 3D. Supplementary Figure S5. Full Western-blotting result of the representative image in Figure 5F.
