# Supplementary material for: Furanocoumarin Notopterol: Inhibition of Hepatocellular Carcinogenesis through Suppression of Cancer Stemness Signaling and Induction of Oxidative Stress-Associated Cell Death
Source: Nutrients. 2023 May 24;15(11):2447. doi: 10.3390/nu15112447 (PMC10255513; doi:10.3390/nu15112447)
Supplement: Supplementary file 1 [file nutrients-15-02447-s001.zip › nutrients-2371533-supplementary.pdf]

Supplementary Materials

Furanocoumarin Notopterol: Inhibition of Hepatocellular Carcinogenesis through Suppression of Cancer Stemness Signaling and Induction of Oxidative Stress-Associated Cell Death

Ting-Yun Huang [1,2,3,†], Ching-Kuo Yang [4,†], Ming-Yao Chen [5,6], Vijesh Kumar Yadav [5,6], Iat-Hang Fong [5,6], Chi-Tai Yeh [5,6,7,*] and Yih-Giun Cherng [8,9,*]

1 Department of Emergency Medicine, Shuang-Ho Hospital, Taipei Medical University, New Taipei City 23561, Taiwan; 19123@s.tmu.edu.tw
2 Graduate Institute of Injury Prevention and Control, College of Public Health, Taipei Medical University, Taipei City 11031, Taiwan
3 Department of Emergency Medicine, School of Medicine, Taipei Medical University, Taipei City 11031, Taiwan
4 Division of Colorectal Surgery, Department of Surgery, Mackay Memorial Hospital, Taipei City 10449, Taiwan; yangchingkao@yahoo.com.tw
5 Division of Gastroenterology and Hepatology, Department of Internal Medicine, School of Medicine, College of Medicine, Taipei Medical University, Taipei City 11031, Taiwan; 08350@s.tmu.edu.tw (M.-Y.C.); vijeshp2@gmail.com (V.K.Y.); 18149@s.tmu.edu.tw (I.-H.F.)
6 Division of Gastroenterology and Hepatology, Department of Internal Medicine, Shuang Ho Hospital, New Taipei City 235041, Taiwan
7 Continuing Education Program of Food Biotechnology Applications, College of Science and Engineering, National Taitung University, Taitung 95092, Taiwan
8 Department of Anesthesiology, Shuang Ho Hospital, Taipei Medical University, New Taipei City 23561, Taiwan
9 Department of Anesthesiology, School of Medicine, College of Medicine, Taipei Medical University, Taipei City 11031, Taiwan
* Correspondence: ctyeh@s.tmu.edu.tw (C.-T.Y.); stainless@s.tmu.edu.tw (Y.-G.C.); Tel.: +886-2-2490088 (ext. 8881) (C.-T.Y.); +886-2-2490088 (ext. 8885) (Y.-G.C.)
† These authors have contributed equally to this work.

Supplementary Table S1. The membranes were incubated in primary antibodies.

No.	Target	Dilution	MW	Source	
1	ATF4	1:1000	50	ab184909	abcam
2	p-JAK2	1:500	125	#3776	Cell signaling
3	JAK2	1:1000	125	#3230	Cell signaling
4	GPX1	1:2000	22	ab108427	abcam
5	Catalase	1:2000	60	ab209211	abcam
6	SOD1	1:2000	23	ab13498	abcam
7	Vimentin	1:2000	57	sc-373717	Santa Cruz
8	Snail	1:1000	29	sc-271977	Santa Cruz
9	β-catenin	1:1000	92	sc-133240	Santa Cruz
10	N-cadherin	1:1000	130	sc-8424	Santa Cruz
11	Oct-04	1:1000	45	sc-5279	Santa Cruz
12	CD133	1:1000	120	sc-365537	Santa Cruz
13	SOX2	1:1000	34	sc-365964	Santa Cruz
14	Bcl-2	1:1000	26	ab32124	abcam
15	PARP1	1:1000	113	ab191217	abcam
16	cleaved PARP1	1:1000	25	ab32064	abcam
17	ALDH1	1:1000	55	#12035	Cell signaling
18	CHOP	1:1000	27	#2895	Cell signaling
19	PERK	1:1000	140	#5683	Cell signaling
20	GAPDH	1:1000	37	sc-32233	Santa Cruz

Supplementary Table S2. List of gene specific primers with detailed sequence used in this study

No.	Target	Primer	Sequence
1	OCT4	F	CCTGAAGCAGAAGAGGATCACC
		R	AAAGCGGCAGATGGTCGTTTGG
2	SOX2	F	GCTACAGCATGATGCAGGACCA
		R	TCTGCGAGCTGGTCATGGAGTT
3	ALDH1	F	CGGGAAAAGCAATCTGAAGAGGG
		R	GATGCGGCTATACAACACTGGC
4	SOD1	F	CTCACTCTCAGGAGACCATTGC
		R	CCACAAGCCAAACGACTTCCAG
5	GPX1	F	GTGCTCGGCTTCCCGTGCAAC
		R	CTCGAAGAGCATGAAGTTGGGC
6	CHOP	F	GGTATGAGGACCTGCAAGAGGT
		R	CTTGTGACCTCTGCTGGTTCTG
7	PERK	F	GTCCCAAGGCTTTGGAATCTGTC
		R	CCTACCAAGACAGGAGTTCTGG

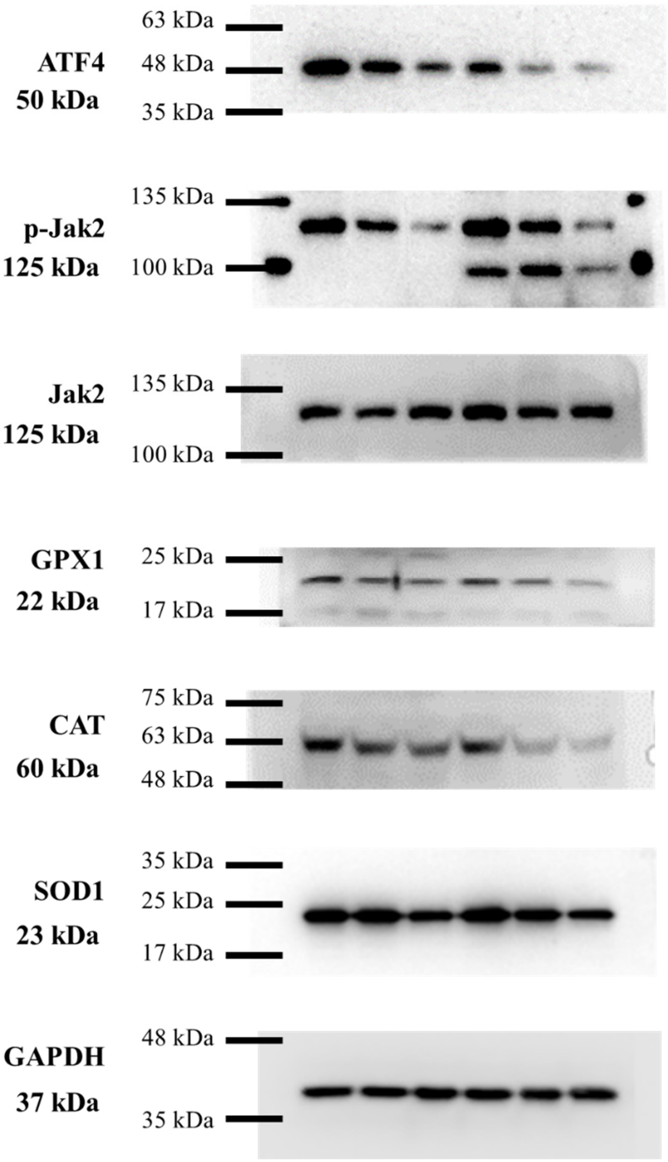

Supplementary Figure S1. Full western-blotting result of representative image in Figure 1D.

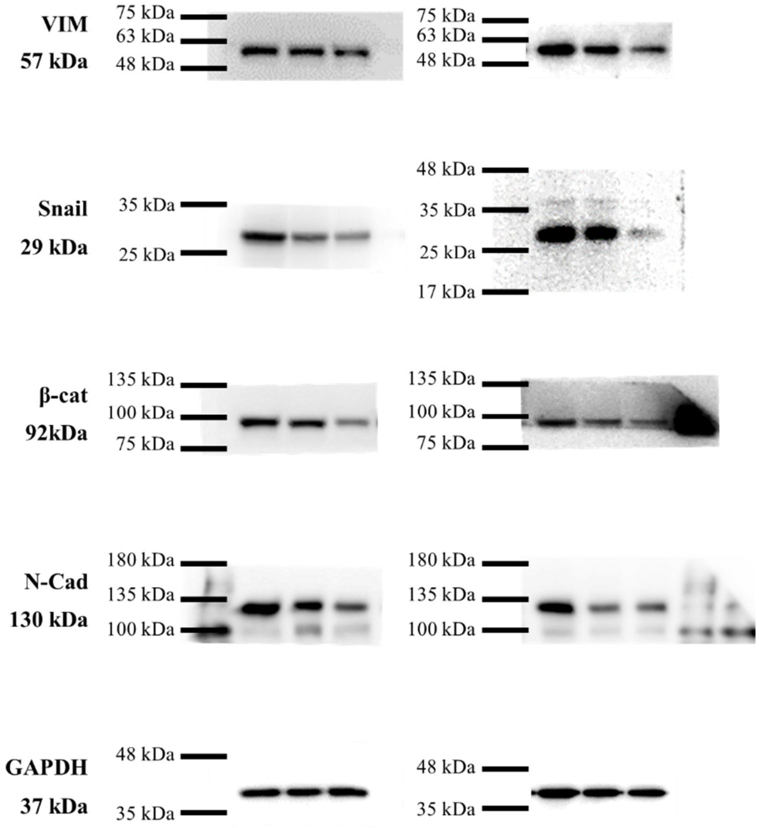

Supplementary Figure S2. Full western-blotting result of representative image in Figure 2C.

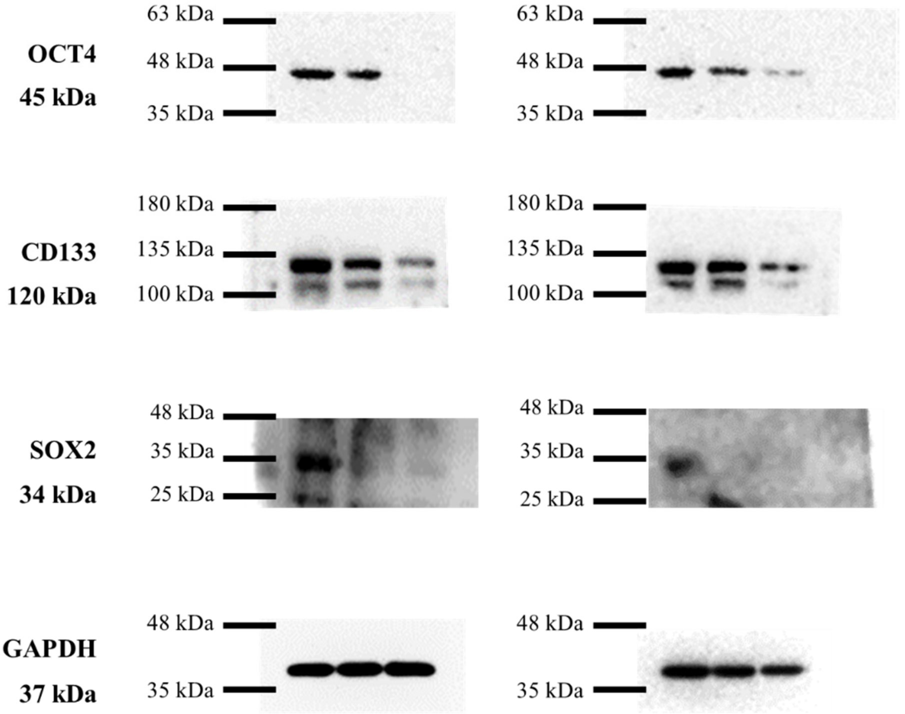

Supplementary Figure S3. Full western-blotting result of representative image in Figure 3C.

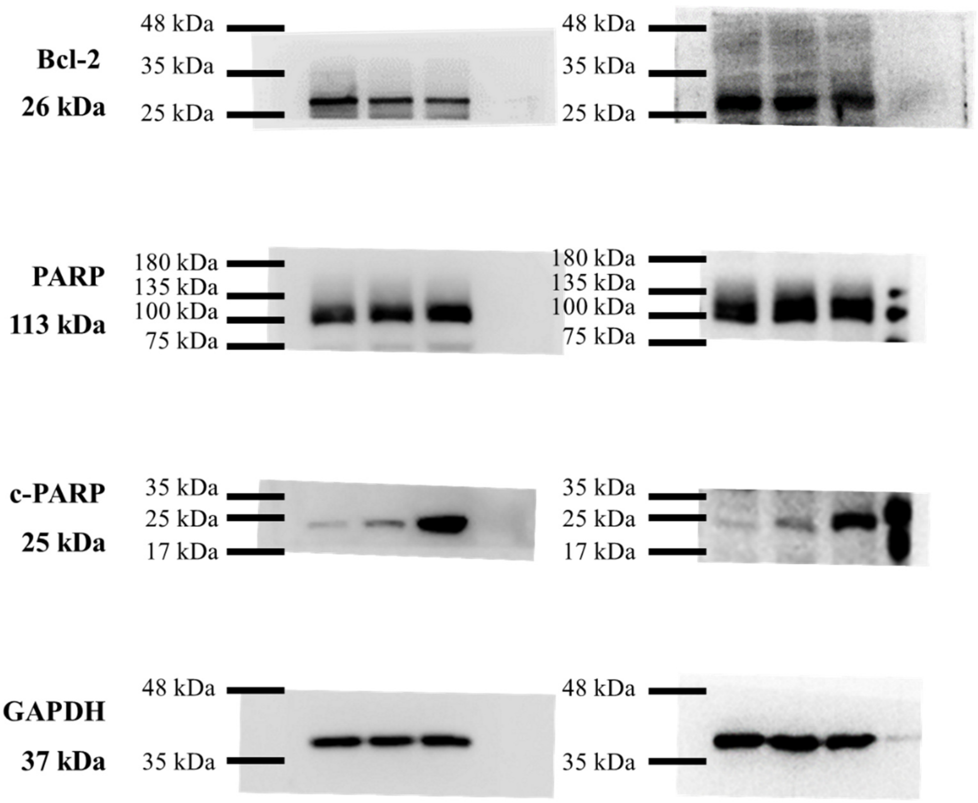

Supplementary Figure S4. Full western-blotting result of representative image in Figure 3D.

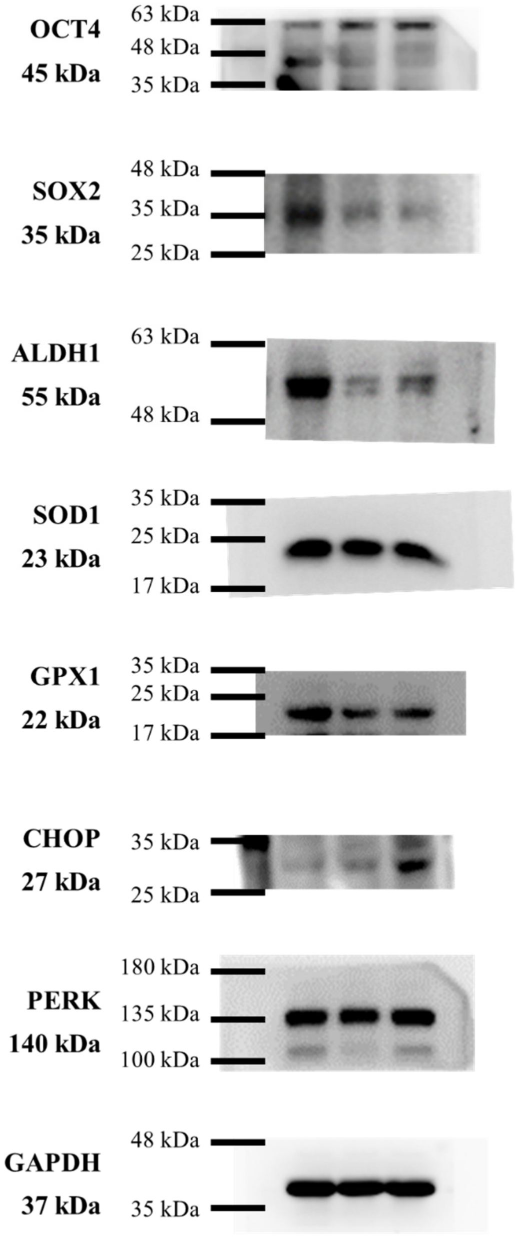

Supplementary Figure S5. Full western-blotting result of representative image in Figure 5F.
